# *Pseudomonas aeruginosa* as an uncommon agent of infectious panniculitis^⋆^

**DOI:** 10.1016/j.abd.2020.09.018

**Published:** 2022-03-07

**Authors:** Tatiana Mina Yendo, Cristina de Castro Pante, Denise Miyamoto

**Affiliations:** Department of Dermatology, Faculty of Medicine, Universidade de São Paulo, São Paulo, SP, Brazil

**Keywords:** Lupus erythematosus, cutaneous, Panniculitis, *Pseudomonas aeruginosa*

## Abstract

*Pseudomonas aeruginosa* is a Gram-negative bacillus that frequently causes septicemia, abscesses and infections in skin wounds. Panniculitis caused by this microorganism is unusual and there are few well-documented cases, none of them in a patient with systemic lupus erythematosus. The present report describes an immunosuppressed patient with systemic lupus erythematosus who developed panniculitis caused by *Pseudomonas aeruginosa*, with a review of the literature on this rare presentation.

## Introduction

*Pseudomonas aeruginosa* (*P. aeruginosa*) is a Gram-negative bacillus that may be the etiological agent of mild to severe skin conditions, such as folliculitis, erysipelas, digital intertrigo, green nail syndrome, ecthyma gangrenosum, and sepsis.^1^ In immunosuppressed and hospitalized patients, *P. aeruginosa* often behaves as an opportunistic pathogen and frequently causes septicemia, abscesses, and wound infections.^2^ Subcutaneous nodules constitute a rare manifestation, and most published case reports did not include a full laboratory investigation, providing limited information on this disease.^3^, ^4^ The present report describes a patient with panniculitis caused by *P. aeruginosa,* with a literature review.

## Case report

**Figure 1:**
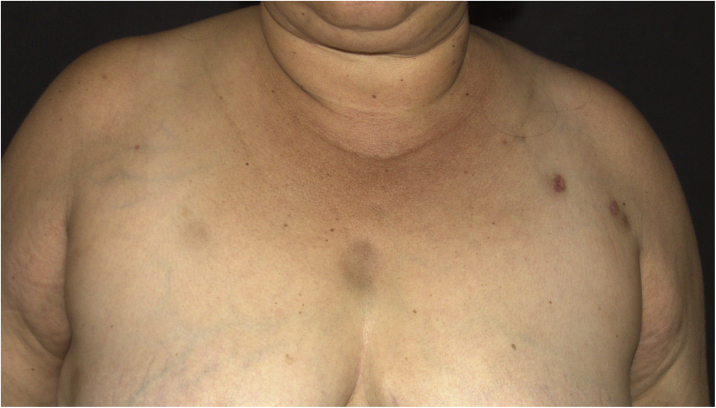
Hyperchromic nodules on the upper thoracic region and left upper limb.

**Figure 2:**
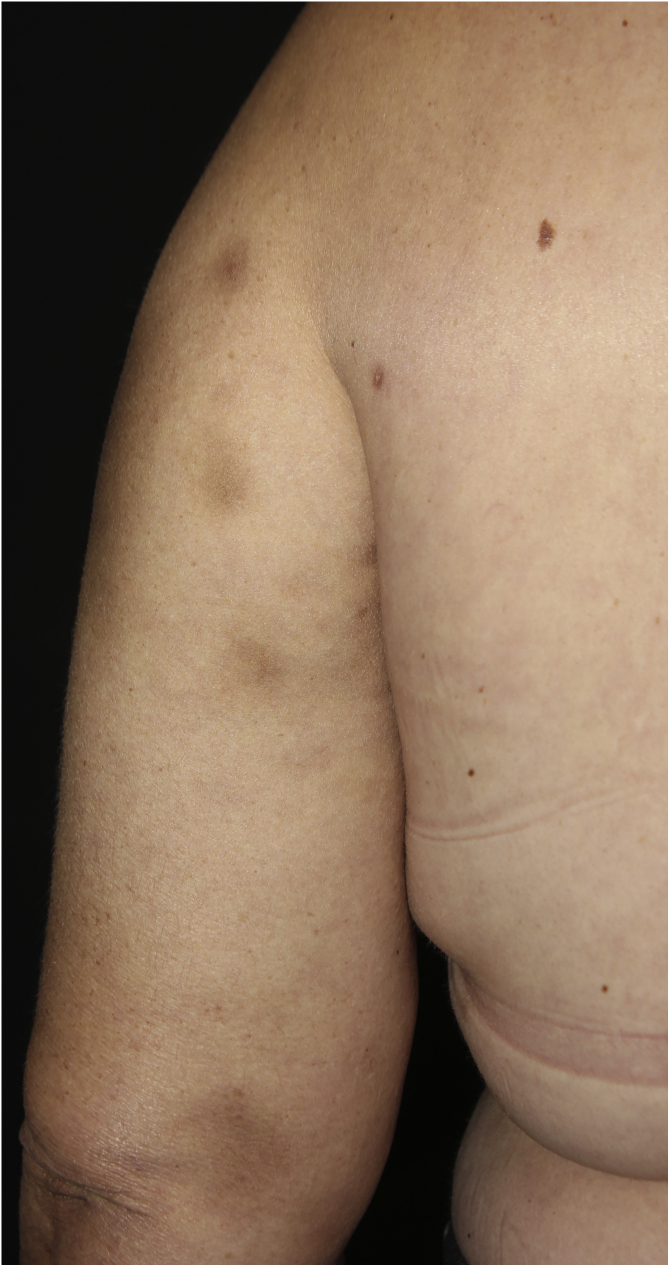
Hyperchromic nodules on the left upper limb.

A 44-year-old female patient, diagnosed with systemic lupus erythematosus (SLE), using prednisone 1 mg/kg/day as an immunosuppressant drug, was admitted to the Rheumatology ward for treatment of gastroenterocolitis and uveitis caused by cytomegalovirus with ganciclovir. During hospitalization, the patient had a *P. aeruginosa* bloodstream infection, which was resolved after treatment with meropenem 2 g every 8 hours for ten days. After one month, a dermatology consultation was requested due to the appearance of erythematous nodules on the upper back, thorax, face, upper limbs and breasts (Figure 1, Figure 2), without other systemic symptoms.

**Figure 3:**
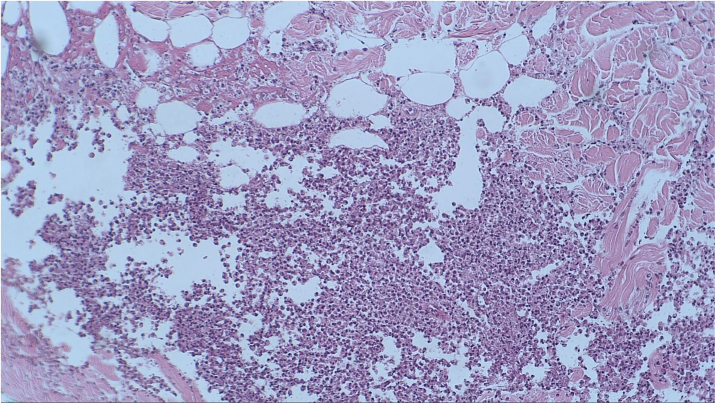
Histopathological examination revealed the presence of a lobular inflammatory infiltrate in the hypodermis (Hematoxylin & eosin, ×100).

**Figure 4:**
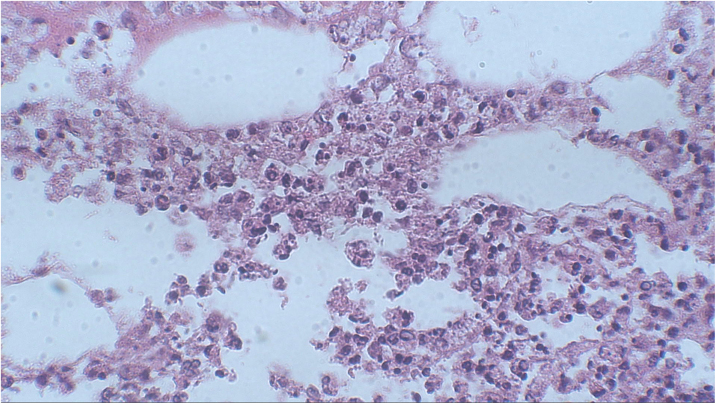
At higher magnification, the histopathological examination revealed the presence of a neutrophilic infiltrate in the hypodermis (Hematoxylin & eosin, ×400).

A punch biopsy was performed on the upper back lesion. The histopathological examination showed a neutrophilic infiltrate in the dermis, associated with suppurative folliculitis that extended to the hypodermis (Figure 3, Figure 4). There were no significant findings in the other exams, including the blood culture.

In the skin culture, *P. aeruginosa* was isolated with an antimicrobial resistance profile identical to that of the bacteria that had been previously obtained in the peripheral blood culture at the time of the bloodstream infection. With these findings, the diagnosis of infectious panniculitis caused by *P. aeruginosa* was confirmed, and after treatment with ciprofloxacin 500 mg, every 12 hours for 4 weeks, the lesions improved without recurrence.

## Discussion

Erythematous nodules on the limbs and trunk can occur in SLE, erythema nodosum, type 2 leprosy reaction, erythema induratum of Bazin, nodular vasculitis, and infectious, traumatic, or insulin-induced panniculitis. Erythema nodosum is the most frequent cause of panniculitis, although in patients diagnosed with SLE, lupus panniculitis or lupus profundus may occur in approximately 1%–3% of these patients.^5^

The anatomopathological examination with neutrophilic infiltrate without vasculitis in the hypodermis is characteristic of pancreatic panniculitis, panniculitis associated to alpha-1-antitrypsin deficiency, and infectious panniculitis.^5^ In infectious panniculitis, the microorganisms can be identified using special staining methods, such as hematoxylin-eosin, Gram or Ziehl-Neelsen, and the identification of the agent is performed through immunohistochemistry, serology or biopsy culture, with the latter being the gold standard for diagnostic confirmation.^6^

*P. aeruginosa* is commonly found in humid environments and in the human intestinal flora. This microorganism can cause both community-acquired and nosocomial skin infections through direct inoculation, hematogenous spread, or intestinal translocation.^1^ Immunosuppression or local alterations in immunity predispose to sepsis, with increased mortality in the hospital environment due to the existence of a multidrug-resistant *P. aeruginosa* strain.^2^

There are only six reports of panniculitis caused by *P. aeruginosa* in the literature with the description of clinical, histopathological, and microbiological diagnoses (Table 1). 7, 8, 9, 10 The patients mean age was 65.1 years (50–80 years), predominantly females (n = 5), and all of them were immunosuppressed (n = 4 over 60 years old, n = 3 with diabetes mellitus, n = 1 with liver cirrhosis, n = 2 undergoing chemotherapy). Regarding the clinical picture, the patients had erythematous nodules, predominantly on the lower limbs, some of which were ulcerated. Three cases had skin lesions accompanied by sepsis, and in two cases, *P. aeruginosa* was isolated from the bloodstream.

**Table 1:** Reported cases of panniculitis caused by *P. aeruginosa* with clinical, histopathological and microbiological confirmation.

Source, year	Age, sex	Dermatological examination	Associated symptoms	Personal history	Anatomopathological examination	Skin culture	Blood culture	Treatment	Outcome
Roriz et al., 2014	80, F	Multiple ulcers on the right lateral malleolus, inflammatory nodules on the left thigh.	Absent	Type I DM, venous insufficiency, CKD	Neutrophilic lobular panniculitis without vasculitis	*P. aeruginosa*	Negative	Ciprofloxacin P.O.	Resolution
Roriz et al., 2014	50, M	Inflammatory nodules on the left lower limb with ulcers secondary to necrotic purpura	Unknown	Type I DM, obesity, pulmonary hypertension and dilated cardiomyopathy	Neutrophilic lobular and septal panniculitis	*P. aeruginosa*	Unknown	Ciprofloxacin P.O.	Resolution
Roriz et al., 2014	70, F	Ulcer on the right lower limb	Unknown	HBV cirrhosis, cardiopathy, venous insufficiency	Lobular and septal panniculitis with intense neutrophilic infiltrate	*P. aeruginosa*	Negative	Ceftazidime and Amikacin IV	Death from liver complications
Penz et al., 2010	72, F	Ulcers on the right lower limb and nodule on the right thigh	Fever	DM, arterial and venous insufficiency, obesity, SAH, HF and CVA	Lobular panniculitis	*P. aeruginosa*	Negative	Cilastatin sodium, imipenem, vancomycin and ciprofloxacin IV	Resolution
Moyano et al., 2011	63, F	Erythematous nodules, some with pustules on the surface	Fever, cough and poor overall status	Microinvasive ductal carcinoma, undergoing CT (cyclophosphamide, adriamycin and docetaxel)	Neutrophilic lobular panniculitis, with abscess and hemorrhage	*P. aeruginosa*	*P. aeruginosa*	Unknown	Unknown
Bagel et al., 1986	56, F	Erythematous subcutaneous nodules, pustules, and hemorrhagic blisters on the extremities	Fever and altered mental status	Metastatic ovarian carcinoma, undergoing CT (cisplatin, cytoxan and adriamycin)	Dense neutrophilic infiltrate in the subcutaneous tissue	*P. aeruginosa*	*P. aeruginosa*	Ticarcillin and Tobramycin IV	Resolution

DM, Diabetes Mellitus; CKD, Chronic Kidney Disease; HBV, Hepatitis B Virus; SAH, Systemic Arterial Hypertension; HF, Heart Failure; CVA, Cerebrovascular Accident (stroke); CT, Chemotherapy; *P. aeruginosa*, *Pseudomonas aeruginosa*; P.O., Oral administration; IV, Intravenous administration.

This is the first report of panniculitis caused by *P. aeruginosa* with confirmatory clinical, histopathological and microbiological examinations in a patient with SLE. As reported, the patient was immunosuppressed and had a previous episode of septicemia caused by *P. aeruginosa*. The hypothesis of the present case is that the patient was colonized by *P. aeruginosa* and that, through hematogenous dissemination, this microorganism reached the hypodermis and triggered the formation of multiple subcutaneous nodules. The importance of considering infectious panniculitis as a differential diagnosis in immunosuppressed patients is emphasized, even in the absence of fever or other signs of sepsis. Early identification and adequate treatment with antibiotics can improve the prognosis of these patients.

## Financial support

None declared.

## Authors' contributions

Tatiana Mina Yendo: Patient follow-up; manuscript preparation.

Cristina de Castro Pante: Patient follow-up; manuscript preparation.

Denise Miyamoto: Manuscript review.

## Conflicts of interest

None declared.
